# Prevalence and Socioeconomic Impact of Allergic Rhinitis Among Ear, Nose, and Throat Patients of a Tertiary Hospital

**DOI:** 10.7759/cureus.49768

**Published:** 2023-12-01

**Authors:** Peter Appiah-Thompson, Amy Amuquandoh

**Affiliations:** 1 Surgery/Otolaryngology, Cape Coast Teaching Hospital, Cape Coast, GHA; 2 Surgery/Otolaryngology, School of Medical Sciences, University of Cape Coast, Cape Coast, GHA; 3 Internal Medicine, Shai Osudoku District Hospital, Dodowa, GHA

**Keywords:** rhinorrhea, sneezing, allergen, immunoglobulin e, hay fever, allergic rhinitis

## Abstract

Objectives: The objectives of this study were to establish the demographic factors of allergic rhinitis patients taking part in the study, to gain insight into the common symptoms experienced by patients with allergic rhinitis, to know the common allergens or triggers that lead to the symptoms of allergic rhinitis and to determine the prevalence of other comorbidities associated with allergic rhinitis.

Methods: The study was a cross-sectional hospital-based study encompassing both quantitative and qualitative features of the participants involved. The quantitative study involved collecting data on allergic rhinitis clients visiting our tertiary hospital, over the period of March 1, 2021, to June 25, 2021. The quantitative data included the gender and age groups commonly affected, the most common symptom, and the trigger identified. Whilst, the qualitative aspect of the study involved the socioeconomic impact of allergic rhinitis on the clients.

Results: The prevalence of allergic rhinitis is 10% at the ENT clinic of our hospital. It was most common in the age group (19-35). Urban residents suffered more from allergic rhinitis than the rural residents. The main presenting complaint was sneezing and the commonest comorbid condition and trigger associated with allergic rhinitis were sinusitis and dust mites respectively.

Twenty-nine percent of respondents had experienced reduced productivity at their workplace and in school. Twenty-one percent had experienced depression while 26% perceived the cost of treatment to be greatly expensive.

The use of face masks was found not to be beneficial in reducing the symptoms of allergic rhinitis in most clients.

Conclusion: The conclusions reached at the end of this study were that the public must be educated on allergic rhinitis and to look out for the triggers, signs, and symptoms of it and then report early to the hospital for appropriate management.

## Introduction

Allergic rhinitis, which is also known as hay fever, is an allergen-induced hypersensitivity response leading to nasal mucosal inflammation [1]. When an individual is exposed to an antigen, in type I hypersensitivity reaction, a specific immunoglobin E (IgE) antibody-mediated process occurs. Histamine and other inflammatory mediators are released following the cross-linking of IgE on the surface of mast cells and basophils. This process leads to the disorders noted in hypersensitivity, for example, allergic rhinitis, urticaria, etc. [2,3].

It is clinically characterized by four main symptoms; namely, rhinorrhoea, sneezing, nasal congestion, and nasal itching [4]. Other symptoms include itchy eyes and throat, coughing, post-nasal drip, sinus pressure, and fatigue. It may be associated with other comorbid conditions like nasal polyps, atopic dermatitis, and asthma [5]. Allergic rhinitis is a systemic airway disease affecting the entire respiratory tract other than just the involvement of the nasal cavity as initially postulated.

The two main types of allergic rhinitis include seasonal allergic rhinitis and perennial allergic rhinitis. Seasonal allergic rhinitis is manifested during certain periods for example during summer or harmattan, hence it occurs at the same time of the year and may be caused by a specific allergen like ragweed, tree, and grass pollen. It is characterized by acute conjunctivitis with itching and lacrimation. The most common manifestation of type I reaction is seasonal allergic rhinitis with a prevalence of at least 25% [3]. Perennial allergic rhinitis may be manifested throughout the year. It is usually due to a reaction to indoor allergens like dust mites, molds, and pet dander. Perennial allergic rhinitis however is more difficult to diagnose since it may overlap with sinusitis, vasomotor rhinitis, and respiratory tract infection [6].

Allergic rhinitis is a common disorder that affects 40% of the world’s population; however, it is usually undiagnosed. Africa and Latin America have the greatest rates of severe allergic rhinitis [5]. This is a result of the geographic variances in the types and potency of different allergens and the global burden of aeroallergen [7].

Africa has witnessed an increase in the incidence of allergic rhinitis. More than 30 million Africans today have allergic rhinitis. These are attributable to migration and movements into urban areas from rural settings. As home settings and lifestyle changes happen, the immunometabolic states also change leading to a general increase in allergic diseases [8]. The International Study on Asthma and Allergy in Childhood (ISAAC) III study in South Africa reported a prevalence of up to 20% of young people between the ages of 13 to 14 years having allergic rhinoconjunctivitis, asthma, and eczema [9]. Another study in Ghana reported a prevalence of 9.1% [10].

Allergic rhinitis is the most common cause of chronic rhinitis and is accompanied by significant impairment in the quality of life, sleep, and work productivity. The socioeconomic impact of allergic rhinitis may be directly or indirectly experienced by the patient involved, the family, society, and the workplace. The direct impact on the individual may be through paying for healthcare services, treatments, and laboratory investigations. Indirectly, patients may be affected due to reduced productivity at their workplaces [11]. Allergic rhinitis was found to cause the greatest workplace productivity loss due to absenteeism etc. In 2005, approximately 11 billion US dollars was estimated to be the direct and indirect costs of allergic rhinitis excluding costs of associated diseases such as sinusitis and asthma [12].

There is however no easily accessible data on the prevalence and socioeconomic impact of allergic rhinitis in Ghana. This research was necessary to enhance the education, diagnosis, and management of allergic rhinitis in Ghana.

## Materials and methods

Study design

The study was a cross-sectional hospital-based study encompassing both quantitative and qualitative features of the participants involved. The hospital setting was used for easy access to allergic rhinitis patients. The quantitative study involved collecting data on both the old and new cases of allergic rhinitis patients visiting our tertiary hospital, over the period of March 1, 2021, to June 25, 2021. The quantitative data thus encompassed the gender and age groups commonly affected, the most common symptom, and the trigger identified.

The qualitative aspect of the study involved the socioeconomic impact of allergic rhinitis on the patients. Data was gathered on how allergic rhinitis affected the patients' quality of life in terms of their social relationships at their various workplaces, and schools, and even in terms of their mental health. A standardized questionnaire containing both closed and open-ended questions was used to collect data at the hospital. The open-ended questions gave respondents the opportunity to express themselves. The primary investigator or the field assistant administered the questionnaires to the subjects. Data was collected over a four-month period from a target client population of 720 and a sample size of 82. The sample size of 82 was calculated using the Cochrane formula with a prevalence rate of allergic rhinitis of 6%. The population size of 720 was chosen based on the average monthly ENT population for the year 2019. This statistic was calculated using secondary data from the hospital for the year 2019, which showed that the monthly average prevalence rate of allergic rhinitis was 6% of the ENT clinic's average monthly population of 720 people.

The University of Cape Coast Institutional Review Board (UCCIRB) approved (UCC/IRB/R/1/1,032) ethical clearance to conduct this research.

Study area

The research took place at our tertiary hospital's ENT clinic, which acts as a referral center for the Central and Western regions, and other adjacent districts of the Ashanti region of Ghana.

Sampling and sampling procedure

The study was a hospital-based cross-sectional study and a convenient sampling method was used to select the sample size based on the inclusion criteria.

Data collection procedures

The data collection process took place on weekdays from March 1, 2021, to June 25, 2021. Between the hours of 8:30 a.m. and 2:30 p.m., the questionnaires were given out in the ENT clinic's waiting room. A questionnaire administration took no more than ten minutes to conduct by a respondent.

In administering the questionnaire, rapport was established with the participants, and the aims of the study were explained to them before the beginning of the interview. All potential respondents were notified of their ability to refuse to participate in the study with no repercussions. Before beginning the interview, secrecy and anonymity were assured once more. The data was collected using a structured questionnaire by the interviewer. The interviewers were the authors or employed field assistants who were nurses at the ENT clinic. The consent of the respondents was sought before proceeding to ask questions pertaining to the study.

If minors were involved, consent was sought from their accompanying guardian. Upon agreeing to participate, the questionnaire was then administered. If the minor was able to comprehend and answer the questions being asked from the questionnaire, the questionnaire was administered to the minor with assistance from the accompanying guardian. If not, the questionnaire was administered to the accompanying guardian on behalf of the minor.

The interviewer recorded the response to the printed questionnaire as the questions were being asked. The questionnaire contained both open and closed-ended questions.

In the case of a non-English-speaking respondent, the questionnaire was translated into Twi or Fante by the interviewer.

All the COVID-19 protocols were strictly adhered to during the data collection process.

Inclusion Criteria

The inclusion criteria for this study included all patients who had nasal symptoms either associated with fever or not or nasal symptoms that subsided and later recurred after a re-encounter with an identified allergen like dust. Other included patients were those who had reported one nasal symptom and either an eye symptom or a specific trigger and those who were aware of having allergic rhinitis.

Exclusion Criteria

The exclusion criteria included patients who qualified for the inclusion criteria but refused to give informed consent and those who did not speak English, or these two local languages, that is, Twi or Fante which would have hampered effective communication during the administration of the questionnaire.

Sample size determination

Convenient sampling was used to select patients for this study at the hospital based on the inclusion criteria.

The sample size, i.e., n = n0/1+ {(n0 -1)/N}, where n0 is Cochran's sample size recommendation and N is the population size.

Before n was calculated, n0 was calculated using Cochran’s equation for the infinite population which is n0=Z(Z)pq/e(e), where e is the desired level of precision (i.e., the margin of error), p is the (estimated) proportion of the population which has the attribute in question and q is 1 - p. The z-value is found in a Z table.

The margin of error (e) = 0.05, confidence interval = 95% and p is the estimated proportion of an attribute that is present in the population, the prevalence. The prevalence p was assumed as 6% (0.06). This was the prevalence of allergic rhinitis in the ENT population obtained from secondary data at the Cape Coast Teaching Hospital, Cape Coast, Ghana.

Thus if p = 0.06, then q=1-p =1-0.06 = 0.94. Z-value for a 95% confidence interval= 1.96. Therefore, n0 = {(1.96)(1.96)(0.06)(0.94)}/(0.05)(0.05) = 86.7.

Now calculating for the sample size n, given the population size (N) of 720 and using the formula n = n0/1+ {(no-1)/N}, the sample size n = 86.7/1+ {(86.7 -1)/720} = 78.

About 5% contingency was assumed. Therefore, the total sample size was set at 82.

Data analysis

The quantitative data analysis was used for the work. Quantitative tools involved tallying and percentage calculations. Data gathered was analyzed using the Statistical Package for Social Sciences (SPSS) version 20 (IBM Corp., Armonk, NY). Tables were drawn with Microsoft Word 2020.

Continuous data were descriptively analyzed using standard deviations and means since the data was regularly distributed. Categorical data were expressed in frequencies and percentages. Frequencies were used to summarize qualitative variables. An estimated prevalence rate of allergic rhinitis was associated with a 95% confidence interval.

Data processing and analysis

Patient anonymity was ensured during the collection of data. The data was entered on a computer with a password only known to the authors. Data collected from the patients on all the questionnaires was entered into SPSS version 20 and crosschecked to ascertain that there were no data errors. After data entry, the filled questionnaires were kept safely in a cabinet under lock and key.

Limitations

There was some incomplete data from some of the respondents however, this was mitigated by increasing the sample size. The exclusion of patients who did not speak English, Twi, or Fante may have impacted our results.

Potential respondents who did not attend the hospital or attended the hospital on weekends were missed in this study.

## Results

A total of 100 patients who matched the inclusion criteria at the ENT clinic were questioned, with 42% being men and 58% being females as shown in Table 1. The mean and modal ages of respondents were 23 years and 25 years respectively with a standard deviation of 16.1. A prevalence rate of 10% was established with a total patient population of 1000 over the four months duration.

**Table 1:** Sociodemographic characteristics

Variable		ƒ (n=100)	%
Sex	Male	42	42.0
	Female	58	58.0
Age (years)	0-10	16	16.0
	11-18	11	11.0
	19-35	52	52.0
	36-50	13	13.0
	51 and above	8	8.0
Place of residence	Urban	67	67.0
	Rural	33	33.0
Occupation	Student	45	45.0
	Teacher	5	5.0
	Health worker	14	14.0
	Skilled worker	19	19.0
	Unskilled worker	7	7.0
	Retired	3	3.0
	None	7	7.0

The majority of the respondents (52.0%) were between the ages of 19-35 years of age, with the modal age being 25 and the youngest and oldest ages being 2 and 81 years respectively (Table 1). The majority of the respondents (67.0%) resided in urban areas while 33.0% resided in rural areas in central, western, and surrounding regions. Study participants were mostly students who made up 45.0% of the respondents (Table 1).

Sneezing was the leading presenting complaint followed by the runny nose, which made up 25.0% and 21.0% of the respondents respectively as shown in Table 2. Some also presented with complications of allergic rhinitis like nasal masses (polyps).

**Table 2:** Presenting complaints, duration of complaints, and triggers of allergic rhinitis

Features		ƒ(n=100)	%
Presenting complaints	Sneezing	25	25.0
	Runny nose	21	21.0
	Sore throat	9	9.0
	Nasal congestion	4	4.0
	Itchy throat	6	6.0
	Headaches	8	8.0
	Nasal mass	5	5.0
	Others, e.g., cough, ear pain, halitosis	22	22.0
Duration of presenting complaints	Less than a month	16	16.0
	1 month to 1 year	29	29.0
	2 to 5 years	23	23.0
	6 to 10 years	15	15.0
	11 to 20 years	17	17.0
Identified triggers	Dust mite	73	73.0
	Pollen	15	15.0
	Animal dander	43	43.0
	Others, i.e., spicy foods, smoke, cold environment	26	26.0

The duration of the presenting complaints had widely varied ranging from a period of 3 days to 20 years (Table 2). However, a majority of the respondents (29.0%) had experienced these symptoms within a period ranging from 1 month to 1 year. The majority of the respondents (73%) identified dust mites as the most common trigger of allergic rhinitis.

Also, for the awareness and knowledge of diagnosis, 38.0% of the respondents had knowledge of their diagnosis (Table 3). The commonest comorbid condition associated with allergic rhinitis from this study was sinusitis which accounted for 20.0% of the respondents while 56.0% had no associated comorbid conditions.

**Table 3:** Knowledge of diagnosis and comorbid conditions

Features		ƒ(n=100)	%
Knowledge of diagnosis	Yes	38	38.0
	No	49	49.0
	Maybe	13	13.0
Comorbid conditions	Asthma or eczema	19	19.0
	Nasal polyps	5	5.0
	Sinusitis	20	20.0
	None	56	56.0

Some respondents (29.0%) had greatly reduced productivity at work, home, and school (Table 4). Those who greatly experienced poor mental concentration at school and at work and memory loss were 22.0% and 18.0% respectively. Among respondents, 24%, 23%, and 31.0% had greatly experienced limited outdoor life, tiredness, and irritability respectively. Also, 21.0% were greatly depressed and 26.0% of the respondents perceived the cost of treatment of allergic rhinitis to be greatly expensive.

**Table 4:** Socioeconomic impact of allergic rhinitis

Variables		Frequency	Percentage
Reduced productivity at work, home, or school	No	25	25.0
	Yes Slightly	46	46.0
	Yes Greatly	29	29.0
Poor mental concentration	No	37	37.0
	Yes Slightly	41	41.0
	Yes Greatly	22	22.0
Memory loss	No	47	47.0
	Yes Slightly	35	35.0
	Yes Greatly	18	18.0
Limitations of outdoor life, i.e., sports and picnics	No	28	28.0
	Yes Slightly	48	48.0
	Yes Greatly	24	24.0
Reduced contact with friends and family even on phone	No	32	32.0
	Yes Slightly	45	45.0
	Yes Greatly	23	23.0
Tiredness	No	21	21.0
	Yes Slightly	48	48.0
	Yes Greatly	31	31.0
Irritability	No	20	20.0
	Yes Slightly	55	55.0
	Yes Greatly	25	25.0
Depression	No	30	30.0
	Yes Slightly	49	49.0
	Yes Greatly	21	21.0
Cost of treatment is expensive	No	21	21.0
	Yes Slightly	53	53.0
	Yes Greatly	26	26.0

From Table 4, several variables like productivity at work, memory loss, irritability, and depression were assessed. We then evaluated the correlation between the aforementioned variables and the perceived cost of treatment.

The results showed that the independent variable (cost of treating allergic rhinitis) had a significant, moderately positive correlation with the other socioeconomic factors of the respondents. Hence, there was a significant positive correlation between the cost of treating allergic rhinitis and the other socioeconomic factors (r=0.62, p< 0.001) as shown in Table 5.

**Table 5:** Correlation between cost and socioeconomic impact of allergic rhinitis

Variable	Socioeconomic factors	Cost of treatment
Socioeconomic factors	1	0.62 (Sig. 0.000)
Cost of treatment	0.62 (Sig. 0.000)	1

**Figure 1:**
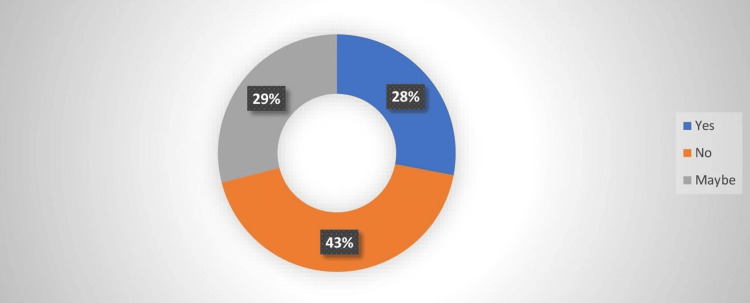
Benefits of the use of face mask in allergic rhinitis

With the advent of the COVID-19 pandemic and the use of face masks, the benefit of the use of face masks in allergic rhinitis was assessed in the study. Some respondents (26.8%) agreed that the use of a face mask is beneficial in helping reduce the symptoms. The majority (43.9%) however disagreed as shown in Figure 1.

## Discussion

The global prevalence of allergic rhinitis is known to be increasing. Globally, its prevalence was 20% in 2018; however, in 2021 it was 10 to 30% in adults and 40% in children [13].

Research conducted at Ekiti State University Hospital, Nigeria, in March 2020 by Adegjbiji et al. established a prevalence rate of 16.4% over a period of one year [14]. Allergic rhinitis prevalence in the United States of America and Europe ranges between 10 to 20% [15].

Despite its increasing prevalence, allergic rhinitis is often underdiagnosed, underestimated, and undertreated. This results in a reduced quality of life in affected individuals [14].

The majority of the respondents (61.0%) in this study resided in urban areas. This finding was similar to a study conducted in Nigeria by Adegbiji et al. where the urban dwellers made up 57.9% and the rural dwellers 42.1% of the respondents [14]. This may be accounted for by the geographic variation in the types and potency of allergens in the urban setting compared to the rural setting as well as their overall burden of aeroallergen [13,14]. The hygiene hypothesis might explain the higher prevalence of allergic rhinitis in urban areas. The hygiene hypothesis suggests that exposure to microbes in early life primes the immune system in the Th1 direction (nonallergic) whilst exposure to a much cleaner environment in early life promotes an exaggerated immune response as noted in allergy [15].

Sneezing was one of the most prevalent symptoms patients with allergic rhinitis reported to the hospital, according to research by Kalpaklioglu et al. [16]. These were similar to the findings in our study. The sneezing reflex is initiated when the nasal cilia are irritated, in this case, by the presence of an allergen. The cilia's receptors transmit signals to the sneeze center in the lateral medulla of the spinal cord via the sensory trigeminal nerves, while other impulses are sent to the parasympathetic nerves to increase tear and nasal secretions. Again, histamine released by mast cells via IgE-mediated inflammatory reaction is mainly responsible for the itchy nose, throat, eyes, and several other symptoms patients experience.

In the research by Kalpaklioglu et al., 33.3% out of 256 were not aware of their diagnosis of allergic rhinitis [16]. The majority of the respondents (49.0%) in our study were also unaware of their diagnosis while 8.6% were unsure of their diagnosis. This further emphasizes the underdiagnosis of allergic rhinitis and hence, a negative socioeconomic impact on the affected patients [16]. 

According to Varshney et al., in 2015, dust mite was among the commonest triggers of allergic rhinitis, similar to the finding in our study [17]. Other triggers included animal dander, pollen, and molds. Other occupational triggers included smoke, nitrogen, and sulfur from automobile exhaust [17].

As found in our study, research conducted in Tanzania at the Muhimbili National Hospital in 2018, sinusitis, asthma, nasal polyps, and eczema were among the common comorbid conditions found in patients with allergic rhinitis patients [18]. These comorbidities increase the morbidity associated with allergic rhinitis. The majority of patients with atopic asthma up to 80% also suffer from allergic rhinitis. The unified airway theory suggests that an allergen challenge of the bronchi results in an inflammatory response in both the bronchi and nasal cavities. Treatment with intranasal corticosteroids thus leads to a decrease in both bronchial and nasal hyperactivity [19].

The findings in our study, i.e., 29.0% of respondents had greatly reduced productivity at work, home, and school were similar to findings in a study performed by Blaiss MS where 10% of full-time workers who took part had missed work in the previous year, and 22% said allergies had affected their productivity and work [20].

When depressed, you are likely to have poor focus, irritability, and fatigue, as well as low energy. In a study, allergic rhinitis was found to be responsible for a 23% drop in workplace productivity [20]. 

The direct and indirect expenses of health care for allergic rhinitis are separated into two groups. The direct cost of health care includes all funds spent in the course of controlling the condition. This includes transportation to medical facilities, consultation fees and other medical services, laboratory and imaging investigations, pharmacological and non-pharmacological therapy as well as management of comorbid conditions and complications associated with allergic rhinitis including surgical cost of treatment of nasal polyps and other procedures [20].

Money wasted due to absence and lower productivity owing to sickness are examples of the indirect or hidden costs of allergic rhinitis. In addition, the monetary worth of time spent caring for a sick child. However, the hidden or indirect cost of allergic rhinitis adds billions of dollars to the annual economic burden. Allergic rhinitis is thus an expensive disease to treat in large populations due to its high prevalence [20].

In research conducted by Dror et al., on the reduction of COVID-19 symptoms with face mask usage among Israeli nurses with allergic rhinitis, it was seen that the use of face masks generally reduced their symptoms [21]. Despite the advantage the N95 respirators have in enhanced small particle trapping over the standard surgical mask, the N95 mask did not have any added advantage over the standard surgical mask in reducing symptoms of allergic rhinitis [21].

Face masks increase the humidity and temperature of expired air between masks and airway orifices which may reduce the nasal responses to provocation by an allergen. Allergens that were not eliminated by face mask filtration may provoke a milder allergic response under face mask-wearing conditions.

Also, since most people wear face masks outdoors, fewer cases of seasonal allergic rhinitis were reported. However, there was an increased indoor allergy caused by dust, mold, and animal dander [21]. This might explain why most. i.e., 43.9% of respondents in our study disagreed with the fact that the face masks were beneficial in helping to reduce symptoms of allergic rhinitis in their lives. This finding is at variance with other studies that have found that the use of face masks reduces both ocular and nasal symptoms of allergic rhinitis [22]. More research in our environment may be needed to clarify this finding.

## Conclusions

The prevalence of allergic rhinitis was 10% at the ENT clinic of our hospital. It was most common in the age group (19-35). Urban residents suffered more from allergic rhinitis than the rural residents. The main presenting complaint was sneezing and the commonest comorbid condition and trigger associated with allergic rhinitis were sinusitis and dust mites respectively.

Twenty-nine percent of respondents had experienced reduced productivity at their workplace and in school. Twenty-one percent had experienced depression while 26% perceived the cost of treatment to be greatly expensive. The use of face masks was found not to be beneficial in reducing the symptoms of allergic rhinitis in most clients though other studies had shown the benefit of face masks in reducing outdoor allergy symptoms.
